# A molecular signature of epithelial host defense: comparative gene expression analysis of cultured bronchial epithelial cells and keratinocytes

**DOI:** 10.1186/1471-2164-7-9

**Published:** 2006-01-18

**Authors:** Joost B Vos, Nicole A Datson, Antoine H van Kampen, Angela C Luyf, Renate M Verhoosel, Patrick L Zeeuwen, Diana Olthuis, Klaus F Rabe, Joost Schalkwijk, Pieter S Hiemstra

**Affiliations:** 1 Dept. of Pulmonology, Leiden University Medical Center, Leiden, Albinusdreef 2, 2333 ZA Leiden, The Netherlands; 2 Dept. of Dermatology, Nijmegen Center for Molecular Life Sciences, Radboud University Nijmegen Medical Center, PO Box 9101, 6500 HB Nijmegen, The Netherlands; 3 Bioinformatics Laboratory, Academic Medical Center, PO Box 22700, 1100 DE, Amsterdam, The Netherlands; 4 Dept. of Medical Pharmacology, Leiden Amsterdam Center for Drug Research, Leiden University Medical Center, Leiden, Einsteinweg 55, 2333 CC Leiden, The Netherlands

## Abstract

**Background:**

Epithelia are barrier-forming tissues that protect the organism against external noxious stimuli. Despite the similarity in function of epithelia, only few common protective mechanisms that are employed by these tissues have been systematically studied. Comparative analysis of genome-wide expression profiles generated by means of Serial Analysis of Gene Expression (SAGE) is a powerful approach to yield further insight into epithelial host defense mechanisms. We performed an extensive comparative analysis of previously published SAGE data sets of two types of epithelial cells, namely bronchial epithelial cells and keratinocytes, in which the response to pro-inflammatory cytokines was assessed. These data sets were used to elucidate a common denominator in epithelial host defense.

**Results:**

Bronchial epithelial cells and keratinocytes were found to have a high degree of overlap in gene expression. Using an *in silico *approach, an epithelial-specific molecular signature of gene expression was identified in bronchial epithelial cells and keratinocytes comprising of family members of keratins, small proline-rich proteins and proteinase inhibitors. Whereas some of the identified genes were known to be involved in inflammation, the majority of the signature represented genes that were previously not associated with host defense. Using polymerase chain reaction, presence of expression of selected tissue-specific genes was validated.

**Conclusion:**

Our comparative analysis of gene transcription reveals that bronchial epithelial cells and keratinocytes both express a subset of genes that is likely to be essential in epithelial barrier formation in these cell types. The expression of these genes is specific for bronchial epithelial cells and keratinocytes and is not seen in non-epithelial cells. We show that bronchial epithelial cells, similar to keratinocytes, express components that are able to form a cross-linked protein envelope that may contribute to an effective barrier against noxious stimuli and pathogens.

## Background

Epithelial tissues in the mammalian airways and skin are among the largest organs and form the interface between the internal milieu of the host and the outside world. They not only protect the host against invading pathogens but also provide an effective barrier to noxious external (chemical and physical) stimuli and dehydration [1,2]. The effectiveness of the epithelial barrier is demonstrated by the rare incidence of severe infections to the lung or skin in healthy individuals. It has become clear that epithelia also play an active role in innate and adaptive immunity [3,4]. Epithelial tissues display three main mechanisms to protect the organism from infection. First, epithelial cells form an impermeable physical barrier which both prevents pathogen entry and minimizes dehydration. Second, epithelial cells are capable of producing defense molecules such as antimicrobial peptides and proteinase inhibitors. Finally, these cells are able to produce signaling molecules such as cytokines and chemokines. These molecules may attract or activate cells of the innate and adaptive immune system [5,6]. Interaction between cells of the immune system is mediated by adhesion molecules and cytokine receptors [7,8] that are present on epithelial cells.

Host defense mechanisms in epithelial cells are coordinated by a complex program of gene expression. Very powerful and sophisticated laboratory techniques such as Serial Analysis of Gene Expression (SAGE) [9] and DNA microarrays [10] have been developed to assess the expression of thousands of genes at the mRNA level in a single experiment. To delineate the barrier function of epithelial cells, the transcriptional change induced by pro-inflammatory cytokines was recently assessed by means of SAGE in two well-established culture models of epithelial inflammation using subcultures of primary bronchial epithelial cells [11] and primary keratinocytes [12]. These independent studies showed a marked overlap in gene families expressed in response to pro-inflammatory cytokines in both cell types. Upon cytokine exposure, in particular genes associated with cytoskeletal architecture and epidermal barrier function such as keratins, S100 calcium-binding proteins and various antimicrobial proteinase inhibitors were differentially expressed. These studies indicated that bronchial epithelial cells and keratinocytes might respond similarly to external influences to ultimately provide effective host protection. This is especially of interest because the epithelia of the skin and conducting airways are markedly different in morphology. The potential functional resemblance of these types of epithelia is also demonstrated by comparative analysis of genetic studies in patients with asthma and atopic dermatitis showing that similar patterns of gene expression may contribute to susceptibility to these diseases [13]. This prompted us to a conduct a comparative analysis of our previously generated gene expression in culture models of epithelial inflammation. The aim was to test the hypothesis whether bronchial epithelial cells and keratinocytes employ similar mechanisms for providing effective host defense at these epithelia.

Therefore, in the present study, our previously generated SAGE data sets derived from bronchial epithelial cells [11] and keratinocytes [12] that were exposed to pro-inflammatory cytokines were compared to identify a common denominator in host defense in the different types of epithelial cells. SAGE libraries of resting and IL1β/TNFα-exposed primary bronchial epithelial cells (~28.000 tags in each library) were compared to SAGE libraries of resting and TNFα-exposed human primary keratinocytes (~13.000 tags in each library). The *in silico *method Tissue Preferential Expression (TPE) [14] was used for the recognition of putative cell-specific gene expression in these SAGE libraries. Previously, this method has been successfully applied to identify novel specific markers for disease [14,15]. To verify the *in silico *prediction analysis of tissue specific gene expression, polymerase chain reaction was performed on seven target genes that were identified by the TPE algorithm in a panel of nine different cell types of which seven are normally present in the airways or lungs. The airway- and lung-derived NCI-H292 and A549 cell lines were included since these cell lines are frequently used to study epithelial cell function. We have identified and validated a signature of specific gene expression for bronchial epithelial cells and keratinocytes. The majority of genes in this signature was previously not associated with host defense or inflammation. These results indicate that epithelia of the airways and skin exploit unified host defense strategies to protect the host, despite their morphological differences.

## Results

**Figure 1:**
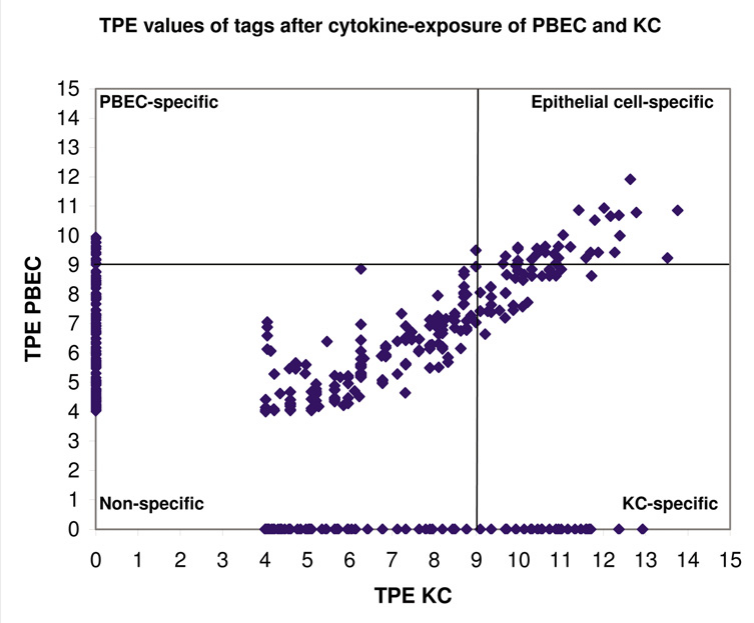
**TPE scatter plot of SAGE tags of PBEC and KC libraries after cytokine exposure. **Reliable 3'-end tags with TPE>4 and tag frequency of = 2 in at least one library were plotted. Tags with corresponding TPE values = 9 in both libraries were considered to be potential epithelial cell-specific tags as indicated by the threshold lines in the figure. A similar picture was obtained when TPE values of tags from the resting libraries were plotted.

Transcriptional overlap between PBEC and KC and epithelial-specific gene expression upon cytokine exposure was characterized. By comparing the four SAGE libraries of primary bronchial epithelial cells (PBEC) and keratinocytes (KC), an overlap in tags of approximately 80% was observed indicating a high similarity in the repertoire of genes expressed by these types of epithelial cells. Although remarkable commonalities were found in gene families found to be expressed by PBEC and KC, the repertoire of transcribed family members differed among the two cell types (table 2). To extract a pattern of genes that is specifically expressed in epithelial cells that could likely be involved in epithelial host defense we explored which of the genes are preferentially expressed by PBEC and KC using the TPE algorithm. The scatter plot in figure 1 displays the individual tags observed in the cytokine-exposed PBEC and KC libraries. Each dot represents a single tag with the corresponding TPE values for PBEC and KC. In this analysis, four groups of tags were identified: epithelial non-specific tags *(i)*, tags preferentially expressed by either PBEC *(ii) *or KC *(iii) *and tags that were preferentially expressed by both PBEC and KC *(iv)*. The expression of the 30 tags observed in the latter group represents putative epithelial-specific genes because a TPE score ≥ 9 was observed in both PBEC and KC (table 3). Almost half of these tags corresponded to genes encoding for keratins, small proline-rich proteins, kallikreins and proteinase inhibitors (table 3). Interestingly, the expression of a large proportion of these genes was found to be affected by cytokine exposure in PBEC or KC (or both) as observed in the initial SAGE studies (as indicated by underlined tag numbers in table 3). A similar picture in preferential tag expression was obtained when using the libraries of resting PBEC and KC since the majority of genes do not show an on/off expression profile upon stimulation with cytokines (data not shown).

**Figure 2:**
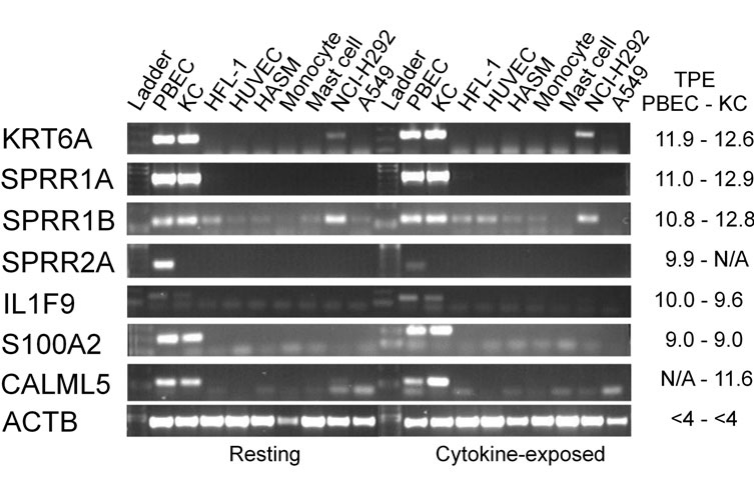
**PCR verification of 7 potential preferentially expressed tags identified using the TPE algorithm**. The expression of these seven genes was assessed both under resting conditions and after cytokine exposure in all cell types. On the right, the TPE values of the gene are listed for both PBEC and KC after cytokine exposure. Tags for which no TPE value could be calculated because of absence of the tag in the particular library are indicated by "not available" (N/A). The predicted preferential expression could be verified for all genes. The expression of six of these genes seems to be selective for epithelial cells only. Whereas SPRR1B is preferentially expressed by epithelial cells, moderate to low levels of expression were also detected in other cell types as well. SPRR2A is preferentially expressed by PBEC only. No tags for SPRR2A were found in the KC libraries after TNFα exposure whereas expression for CALML5 was observed by RT-PCR in both PBEC and KC, while no tags for this gene were found in PBEC libraries.

To validate this *in silico *TPE prediction analysis, expression of seven putative epithelial-specific genes by PBEC and KC was assessed by reverse transcriptase polymerase chain reaction (RT-PCR) in nine different cell types. Each cell type in the panel was exposed to medium alone or to IL1β/TNFα. KC were exposed to medium or TNFα alone to maintain comparability with the original SAGE experiment. In concordance with the SAGE data and TPE analysis, expression for SPRR2A was only observed in PBEC. On the other hand, CALML5 was expected to be expressed by KC alone. However, PBEC were shown to be positive for this transcript as well and weak expression was observed in NCI-H292 cells. As demonstrated by the TPE analysis KRT6A, SPRR1A, SPRR1B, IL1F9, S100A2 all showed TPE values of ≥ 9 in both PBEC and KC libraries. The RT-PCR results in figure 2 demonstrates that preferential expression of SPRR1B was found in PBEC, KC and NCI-H292 cells, whereas moderate to weak expression was also detected in fibroblasts, HUVEC, HASM and monocytes. Expression of KRT6A is restricted to PBEC, KC and the bronchial epithelial cell line NCI-H292, whereas expression of this transcript was negative in all other cell types. Transcription of SPRR1A, IL1F9 and S100A2 was only detected in primary cultures of PBEC and KC and was completely absent in all other cell types.

## Discussion

Comparative genomics approaches have the potential to gain additional insight into a biological process at the mRNA expression level by integrating and combining data obtained from similar model systems. Particularly, SAGE is excellent for this purpose since digital, scalable expression data is generated that allows comparison without the need for complex mathematical normalization methods. Although the SAGE libraries used in the present analysis were not initially intended for comparative genomic research, remarkable commonalities in epithelial-specific gene expression were found that related to host defense.

The tissue preferential expression (TPE) algorithm was employed to recognize specific tag expression by PBEC and KC under inflammatory conditions (pane iv; figure 1, table 3). Experimental verification of selected epithelial-specific genes by RT-PCR showed a good correlation between the *in silico *approach and RT-PCR (figure 2). The PCR setup was designed to detect true presence or absence of validation genes and was not intended to be quantitative. The observed discrepancies between SAGE and PCR results can be explained by the difference in detection sensitivity between techniques: RT-PCR is far more sensitive than SAGE in detecting low abundant gene expression.

The majority of tags of the molecular signature corresponded to genes encoding structural components of the cytoskeleton (keratins, small proline-rich proteins, elafin) and for proteins that are involved in the assembly/disassembly (transglutaminase 1, kallikreins and matrixmetalloproteinases) of the cornified cell envelope in keratinocytes (reviewed in [16]). Components of the cross-linked or cornified envelope are linked by transglutaminases (reviewed in [17]). The observation that bronchial epithelial cells express components of and assembly/disassembly enzymes forming a cross-linked envelope is relevant to our understanding of epithelial host defense in the airways. Additional support for this observation is provided by abundant transcription of genes that are known to be involved in cornification in skin, including the S100 calcium-binding proteins [18], annexins [18] and cystatins [19,20] (table 2). So far, only few studies provide evidence for the existence of a protein envelope in bronchial epithelial cells. Components such as small proline-rich proteins (SPRR) have been suggested to be associated with squamous differentiation [21,22]. Low SPRR expression has been associated with squamous cell carcinoma [23], whereas high expression of SPRR1B enhanced G0-arrest resulting in growth arrest [24]. Interestingly, the families of small proline-rich proteins and S100 calcium-binding proteins are encoded in the epidermal differentiation complex (EDC) [25,26]. Proteins encoded in this region share significant sequence similarities, particularly in the glutamine- and lysine-rich regions that are involved in the cross-linking by transglutaminases [17]. This indicates that PBEC and KC not only share structural characteristics, but may also share functional characteristics.

A disadvantage of the present study might be the differences in type of cytokine-exposure and duration of the treatment. The opposite directional changes in expression in gene families (table 2) observed could be explained either by dissimilarities in the initial model systems or by the inherent differences between PBEC and KC. By using the TPE algorithm, highly cell-specific tag expression can be predicted largely independently from transcriptional levels because the more unique a tag is to a particular tissue, the less important is its level of expression. Therefore, we are confident that the signature of epithelial host defense that was extracted is representative for bronchial epithelial cells and keratinocytes.

Computational subtraction methods such as the TPE algorithm allow functional clustering of genes derived from large and complex genome-wide expression profiles without having full knowledge of the repertoire of genes involved in biological processes of interest. Although the identified molecular signature of host defense is characteristic for bronchial epithelial cells and keratinocytes, it would be of great interest to study whether this gene expression pattern is also applicable to other types of epithelial cells, a finding that would greatly enhance our understanding of epithelial defense strategies.

## Conclusion

In summary, our comprehensive comparison of overlapping genes across bronchial epithelial cells and keratinocytes provides novel insights in epithelial host defense strategies, in particular of the airway epithelium. Combining *in silico *and experimental approaches is very valuable in accelerating the interpretation of genomics data and defining follow-up research. We identified an expression signature of genes that were specifically expressed by bronchial epithelial cells and keratinocytes. These genes are likely to fulfill an eminent function in epithelial host defense. Based on the present findings we propose that formation of a cross-linked protein envelope by bronchial epithelial cells is an effective host defense strategy of the mucosal epithelium in the human airways. This function would be analogous to the host defense function of cornifying keratinocytes. Finally, a better understanding of unified host defense strategies in different epithelia may lead to the identification of novel therapeutic targets for epithelial inflammatory disorders such as asthma and atopic dermatitis.

## Methods

### SAGE data

The previously published SAGE libraries that were compared in this study were derived from two models of epithelial inflammation using primary bronchial epithelial cells [11] and primary keratinocytes [12]. The SAGE data from the original studies is accessible through NCBI's Gene Expression Omnibus [27] with GEO accessions GSM37337 (PBEC_unstimulated), GSM37339 (PBEC_IL1beta/TNFalpha), GSM1121 (NormCultKC_Diff) and GSM1122 (TNF_AlphaCultKC).

For tag mapping, after discarding tags occurring only once, the libraries were compared with NCBI's "reliable Unigene cluster to SAGE tag map" [28] and with SAGEgenie of the Cancer Genome Anatomy Project [29]. Both maps were based on Unigene build#171. Additionally, to enhance the reliability of tag identity we included the virtual tag classification as used in SAGEgenie to assess the location of each tag within the corresponding transcript. Reliable tags can be discriminated from tags that are not isolated from the 3'-end such as internally primed transcripts and tags derived from internal NlaIII restriction sites [29,30]. In the data set comparisons, only tags were included that were derived from the most 3'- restriction site of NlaIII, tags that matched to undefined 3'-end transcripts and tags for which no additional information was available. The last category may contain tags that correspond to novel transcripts.

### TPE analysis

Epithelial-specific gene expression in PBEC and KC was identified using the Tissue Preferential Expression (TPE) algorithm [14]. The calculated Tissue Preferential Expression (TPE) value is based both on the presence of a particular tags and its level of expression in the SAGE library of interest in comparison to a panel of reference SAGE libraries derived from a range of different whole tissues. To allow calculation of TPE values, each of the PBEC and KC SAGE libraries as well as the reference libraries were normalized to a equal number of tags per library. TPE values were determined for each tag in the individual PBEC and KC libraries by selecting each of the libraries as library of interest before applying the TPE algorithm. After calculation, the TPE values were ranked according to their value. Large positive TPE values represent tissue-specific genes that are overexpressed in the PBEC and/or KC libraries. Tags with TPE values of <4 were excluded from further analysis since these tags occur very frequently in other cell types as well (See supplement for a detailed description of the TPE algorithm). The threshold value for the TPE analysis that is indicative for tissue-specific expression was chosen very high to prevent possible false positives. Tags with a corresponding TPE value of ≥ 9 is indicative for tissue preferential expression in the epithelial cells used in this analysis. See additional file for a detailed description of the TPE algorithm.

### Cell culture

#### Primary Bronchial Epithelial Cells (PBEC) and Primary Keratinocytes (KC)

Subcultures of human primary bronchial epithelial cells and human primary keratinocytes were derived and cultured as described previously [31,32].

#### A549 and NCI-H292 cells

the lung derived epithelial cell lines A549 (CCL-185, American Type Culture Collection, Rockville, MD, USA) and NCI-H292 (CRL-1848, American Type Culture Collection) were cultured according to the supplier's recommendation. Prior to stimulation, cells were cultured overnight in serum free medium.

#### Human airway smooth muscle cells (HASM)

Human airway smooth muscle cells (HASM) from two donors were purchased from Stratagene (La Jolla, CA) and were cultured as described previously [33].

#### Human mast cells (HMC-1)

HMC-1 were kindly provided by J.H. Butterfield [34] and were cultured in IMDM medium containing 25 mM Hepes, 2 mM L-glutamine, 20 U/ml penicillin, 20 μg/ml streptomycin (all from Bio Whittaker, Walkersville, MD), 5 μg/ml apo-transferrin, 0,36% β-mercaptoethanol and 10% heat-inactivated Fetal Calf Serum (FCS; GibcoBRL/Life Technologies, Breda, The Netherlands). Prior to stimulation, cells were cultured overnight in serum free medium (same as above, without heat-inactivated FCS)

#### Human lung fibroblasts (HFL-1)

HFL-1 (CCL-153, American Type Culture Collection) were cultured according to the supplier's recommendations. Prior to stimulation, cells were cultured overnight in serum free medium.

#### Monocytes

CD14-purified monocytes were kindly provided by the department of Nephrology (Leiden University Medical Center, Leiden, The Netherlands), and were resuspended in RPMI 1640 medium supplemented with 20 U/ml penicillin, 20 μg/ml streptomycin, 2 mM glutamine (all from Bio Whittaker) and 10% heat-inactivated FCS and were seeded in 6-wells plates to allow adherence. After 2 hours, the medium was replaced by serum free medium (same as above, without heat-inactivated FCS) for overnight incubation prior to stimulation.

#### Human umbilical vein endothelial cells (HUVEC)

HUVEC were kindly provided by the department of Nephrology, Leiden University Medical Center, Leiden, The Netherlands. All cell cultures were performed at 37°C, 5% CO_2 _and 95% relative humidity.

### Stimulation of cell cultures

All cell types, except for the keratinocytes, were stimulated for 6 hours with either medium alone, a mixture of the pro-inflammatory cytokines IL1β (20 ng/ml; PeproTech, Rocky Hill, NJ) and TNFα (20 ng/ml; PeproTech). Keratinocytes were stimulated with TNFα (25 ng/ml) for 48 hours as described previously [12].

### Reverse Transcription PCR

RT-PCR was used to verify preferential expression of genes identified in the TPE analysis. Total RNA from the nine different cell cultures was extracted using the RNeasy mini kit (Qiagen, Westburg, Leusden, The Netherlands) and on-column DNA digestion was performed with DNase I (Qiagen, Westburg), all according to the manufacturer's instructions. Single-stranded cDNA was synthesized of total RNA using M-MLV Reverse Transcriptase primed with Oligo-dT (both from Invitrogen/Life Technologies, Breda, The Netherlands) in the presence of a RNase inhibitor (RNaseOUT; Invitrogen/Life Technologies) according to the manufacturer's instructions. Gene-specific primers were designed for keratin 6A (KRT6A), small proline-rich protein and 1B (SPRR1B), the calcium-binding protein S100A2, IL1 family member 9 (IL1F9), calmodulin-like 5 (CALML5) and β-actin (ACTB) as internal control (table 1). Primers for the small proline-rich protein 1A and 2A [35] were kindly provided by Claude Backendorf. Primers were synthesized by Isogen (Maarssen, The Netherlands). All PCR reactions were carried out according to the following PCR conditions: initial denaturation of 3 minutes at 95°C, then 35 cycles of 15 seconds denaturing at 95°C, 15 seconds primer annealing, 30 seconds elongation at 72°C, and a final extension of 3 minutes at 72°C in the last cycle. For β-actin, the cycle number was limited to 25. PCR products were visualized on 1% agarose gels using ethidium bromide. Both the reverse transcription and PCR reactions were performed on a Biometra T-Gradient thermocycler (Biometra GmbH, Goettingen, Germany).

## Authors' contributions

JBV was primarily responsible for study design and coordination, building comparative SAGE expression databases, performing computational analyses and drafting the manuscript. NAD and PSH participated in study design, coordination and manuscript editing. AHvK and ACL advised in study design and performed the TPE computational analyses. DO, JS and PLZ provided the keratinocyte SAGE data, supplied samples and reagents and assisted in editing the manuscript. RMV performed cell culture experiments, PCR analyses and provided comments and discussion. KFR provided useful discussion and manuscript editing. All authors read and approved the final manuscript.

## Supplementary Material

Additional File 1 A detailed description of the Tissue Preferential Algorithm has been provided as supplemental material in a Word-document entitled "Materials supplement.doc". Click here for file

## Figures and Tables

**Table 1:** Primer sequences and conditions for RT-PCR. Gene sequences used for primer design were retrieved from the Ensembl website [36], annealing temperature and MgCl_2 _concentration used in PCR reactions are listed.

**Target**	**Ensembl ID**	***Sense***	***Antisense***	**Annealing T**	**MgCl_2 _(mM)**
KRT6A	ENSG00000074729	5'-CTG AGG CTG AGT CCT GGT AC-3'	5'-GTT CTT GGC ATC CTT GAG G-3'	56	2.5
SPRR1A	ENSG00000169474	5'-ACA CAG CCC ATT CTG CTC CG-3'	5'-TGC AAA GGA GCG ATT ATG ATT-3'	52	2
SPRR1B	ENSG00000169469	5'-AGA CCA AGC AGA AGT AAT GTG-3'	5'-AGA CCT TCA GCT TCA TTC AGA G-3'	61	4
SPRR2A	ENSG00000163212	5'-TGG TAC CTG AGC ACT GAT CTG CC-3'	5'-CCA AAT ATC CTT ATC CTT TCT TGG-3'	58	2
IL1F9	ENSG00000136688	5'-TGG GAA TCC AGA ATC CAG-3'	5'-TTG GCA CGG TAG AAA AGG-3'	61	3.5
S100A2	ENSG00000160675	5'-CAA GAG GGC GAC AAG TTC-3'	5'-GCC CAT CAG CTT CTT CAG-3'	59	3
CALML5	ENSG00000178372	5'-GGT TGA CAC GGA TGG AAA CG-3'	5'-AAC CTC GGA GAT GAG TTT CCT TAG-3'	60	3
ACTB	ENSG00000075624	5'-AAG GAA GGC TGG AAG AGT GC -3'	5'-CTA CAA TGA GCT GCG TGT GG -3'	56	2

**Table 2:** Inventory of gene families and their members expressed by PBEC and/or KC.

	**SAGE Tag count**^#^						**Tissue Preferential Expression values**			
**SAGE Tag Sequence**	**PBEC CTRL**	**PBEC IL1β/TNFα **	**KC CTRL**	**KC TNFα **	**Symbol**	**Description**	**PBEC CTRL**	**PBEC IL1β/TNFα **	**KC CTRL**	**KC TNFα **

**Keratins**										
ACATTTCAAA	0	0	145	127	KRT1	keratin 1	*	*	13,4	12,9
GCCCCTGCTG	96	180	108	115	KRT5	keratin 5	10,2	10,9	11,4	11,6
AAAGCACAAG	267	252	136	235	KRT6A	keratin 6A	12,0	11,9	12,3	12,6
CGAATGTCCT	21	60	84	97	KRT6B	keratin 6B	9,7	10,7	12,2	12,3
GATGTGCACG	12	26	419	514	KRT14	keratin 14	8,6	9,2	13,5	13,5
CAGCTGTCCC	21	25	26	29	KRT16	keratin 16	9,9	10,0	11,1	11,3
CTTCCTTGCC	181	216	431	380	KRT17	keratin 17	9,8	10,0	12,7	12,4
GACATCAAGT	20	48	0	0	KRT19	keratin 19	5,6	6,5	*	*
**Small proline-rich proteins**										
CTGTCACCCT	9	25	126	75	SPRR1A	small proline-rich protein 1A	10,3	11,1	13,8	12,9
CCCTTGAGGA	27	56	264	176	SPRR1B	small proline-rich protein 1B	10,1	10,8	13,5	12,8
ATGATCCCTG	3	12	2	0	SPRR2A	small proline-rich protein 2A	9,1	9,9	10,4	*
TTTCCTGCTC	91	75	0	2	SPRR3	small proline-rich protein 3	9,1	8,9	*	6,3
**Calcium binding proteins**										
GATCTCTTGG	123	170	103	113	S100A2	S100 calcium binding protein A2	8,5	9,0	9,0	9,0
CCCCCTGGAT	61	133	82	32	S100A6	S100 calcium binding protein A6	<4	4,1	<4	<4
TACCTGCAGA	139	214	28	77	S100A8	S100 calcium binding protein A8	5,8	6,4	4,2	5,5
GTGGCCACGG	121	419	77	156	S100A9	S100 calcium binding protein A9	5,6	7,3	6,2	7,2
AGCAGATCAG	114	86	35	70	S100A10	S100 calcium binding protein A10	<4	<4	<4	<4
CAGGCCCCAC	15	29	9	11	S100A11	S100 calcium binding protein A11	<4	<4	<4	<4
TGGGGAGAGG	53	51	30	32	S100A14	S100 calcium binding protein A14	7,7	7,7	8,7	8,7
AGCAGGAGCA	26	54	40	18	S100A16	S100 calcium binding protein A16	4,3	5,3	5,2	4,2
ATCCGCGAGG	0	0	30	29	CALML5	calmodulin-like 5	*	*	12,0	11,6
**Annexins**										
AGAAAGATGT	102	62	7	7	ANXA1	annexin A1	4,5	<4	<4	<4
CTTCCAGCTA	16	43	77	109	ANXA2	annexin A2	<4	<4	4,5	5,0
AAGGGCGCGG	4	8	12	0	ANXA3	annexin A3	<4	4,2	4,7	*
TTGTTATTGC	5	0	2	5	ANXA7	annexin A7	<4	*	<4	<4
CCCTCAGCAC	3	2	0	14	ANXA8	annexin A8	7,9	7,6	*	9,9
**Proteinase inhibitors**										
ATCCTTGCTG	94	85	150	91	CSTA	cystatin A	8,7	8,6	10,6	9,9
ATGAGCTGAC	64	135	37	38	CSTB	cystatin B	5,0	6,1	4,1	4,1
GTGGAGGGCA	2	2	12	27	CST6	cystatin E/M	4,7	4,7	6,6	7,3
CATTGTAAAT	16	12	26	14	SERPINB5	serine proteinase inhibitor, member 5	9,6	9,4	11,3	10,4
TTGAATCCCC	30	80	56	50	PI3	elafin, protease inhibitor 3, (SKALP)	8,0	9,0	9,9	9,6
TGTGGGAAAT	18	40	101	124	SLPI	secretory leukocyte protease inhibitor	5,3	6,3	7,7	8,0

Expression of these gene families was observed in both PBEC and KC, whereas these cells differ in the expression pattern of the individual family members. From left to right the SAGE tag sequence, SAGE tag counts as observed in the SAGE libraries (unstimulated PBEC, IL1β/TNFα-stimulated PBEC, unstimulated KC and TNFα-stimulated KC), HUGO approved gene symbol and gene description and the calculated Tissue Preferential Expression Values (unstimulated PBEC, IL1β/TNFα-stimulated PBEC, unstimulated KC and TNFα-stimulated KC) are indicated. No TPE values could be calculated for those tags that were absent in one or more libraries and are indicated by (*) in the table. ^#^A selection of these SAGE tag counts were represented in previously published tables [11,12]; all tag counts are available online through the NCBI Gene Expression Omnibus (GEO) website [37] with GEO accessions as listed in the methods.

**Table 3:** 30 Epithelial-specific genes as identified by the TPE analysis.

	**SAGE Tag count**^#^						
**SAGE Tag Sequence**	**PBEC CTRL**	**PBEC IL1β/TNFα **	**KC CTRL**	**KC TNFα **	**Symbol**	**Description**	**Barrier formation**
AAAGCACAAG	267	252	136	235	KRT6A	keratin 6A	***yes***
CTTCCTTGCC	181	216	431	380	KRT17	keratin 17	***yes***
GCCCCTGCTG	96	180	108	115	KRT5	keratin 5	***yes***
TAAACCTGCT	40	68	758	439	LGALS7	lectin, galactoside-binding, soluble, 7 (galectin 7)	
TTGAATCCCC	30	80	56	50	PI3	protease inhibitor 3, skin-derived (SKALP), Elafin	***yes***
CCCTTGAGGA	27	56	264	176	SPRR1B	small proline-rich protein 1B (cornifin)	***yes***
CGAATGTCCT	21	60	84	97	KRT6B	keratin 6B	***yes***
CAGCTGTCCC	21	25	26	29	KRT16	keratin 16	***yes***
AGCTTCTACC	17	22	14	25	HCG9	HLA complex group 9	
CATTGTAAAT	16	12	26	14	SERPINB5	serine (or cysteine) proteinase inhibitor, clade B (ovalbumin), member 5	
GATGTGCACG	12	26	419	514	KRT14	keratin 14	***yes***
CTGTCACCCT	9	25	126	75	SPRR1A	Small proline-rich protein 1A	***yes***
CCCTGTTGAT	8	8	7	18	KLK7	kallikrein 7 (chymotryptic, stratum corneum)	
GGCTTCTAAC	4	16	35	38	SPRR2B	small proline-rich protein 2B	***yes***
GAAGCACAAG	4	13	9	18		Transcribed sequences	
CCAGCGCCAA	3	16	16	14	C4.4A	GPI-anchored metastasis-associated protein homolog	
GCTTCCTCGG	2	11	2	5	RHCG	Rhesus blood group, C glycoprotein	
TCTCTTGGGG	2	4	0	2	FLJ11036	hypothetical protein FLJ11036	
AAAGCACAAT	1	1	0	5	TRA1	tumor rejection antigen (gp96) 1	
TCCTGGATCA	1	1	0	2	KLK10	kallikrein 10	
ATCCCTTGCT	1	2	9	2		Transcribed sequences	
AGAGCACAAG	1	1	2	5		Transcribed sequences	
CTTGCCTTGC	0	2	0	2	ZDHHC9	zinc finger, DHHC domain containing 9	
ACCTCCACTG	0	2	54	34	UNQ467	KIPV467	
CTGCTCAATG	0	3	9	11	TGM1	transglutaminase 1	***yes***
TTCCCTTACC	0	2	5	16	SPRL6A	small proline rich-like 6A	***yes***
ACCTGGAGGG	0	12	28	23	PCBP1	poly(rC) binding protein 1	
GGGCCACGGC	0	1	7	14	MMP11	matrix metalloproteinase 11 (stromelysin 3)	
ACTAGCACAG	0	7	0	2	IL1F9	interleukin 1 family, member 9	
TAGACCTGCT	0	3	2	2		CDNA FLJ32217 fis, clone PLACE6003771	

Putative epithelial-specific tags as presented in pane iv of the scatter plot in figure 1 as identified by the TPE analysis are listed in table 3. From left to right are indicated the SAGE tag sequence, the normalized SAGE tag counts (unstimulated PBEC, IL1β/TNFα-stimulated PBEC, unstimulated KC and TNFα-stimulated KC), HUGO approved gene symbol and gene description and the putative involvement in epithelial barrier formation. Underlined tag counts indicate statistical significant difference in expression in the original SAGE data sets of PBEC (unstimulated vs. IL1β/TNFα) or KC (unstimulated vs. TNFα). ^# ^A selection of these SAGE tag counts were represented in previously published tables [11,12]; all tag counts are available online through the NCBI Gene Expression Omnibus website [37] with GEO accessions as listed in the methods.
