# Supplementary material for: A molecular signature of epithelial host defense: comparative gene expression analysis of cultured bronchial epithelial cells and keratinocytes
Source: BMC Genomics. 2006 Jan 18;7:9. doi: 10.1186/1471-2164-7-9 (PMC1382211; doi:10.1186/1471-2164-7-9)
Supplement: Additional File 1 — A detailed description of the Tissue Preferential Algorithm has been provided as supplemental material in a Word-document entitled "Materials supplement.doc". [file 1471-2164-7-9-S1.doc]

***Materials supplement***

***Detailed description of the Tissue Preferential Expression Algorithm***

The identification of epithelial-specific gene expression in PBEC and KC is facilitated through the calculation of the so-called Tissue Preferential Expression (TPE) values [14]. This TPE is a scalar value that is based on the actual expression levels observed in a selection of SAGE libraries and on the number of SAGE libraries in which a specific gene is observed. This implies that the tissue specificity of a gene is not only determined by counting the number of tissues (SAGE libraries) where a gene is expressed, but that also its expression level plays a role. In particular, a gene that is expressed in multiple tissues but has a much higher expression in the reference tissue would still be denoted as tissue specific. Prior to the TPE calculation a selection of *N* SAGE libraries was made and these libraries were normalized to give equal total tag counts in each library to allow their comparison. One library was selected as the library of interest for which tissue-specific genes were determined. For the current application we selected 15 SAGE libraries that were generated from normal human tissues (brain white matter, prostate, cerebellum, ovary, brain, leukocyte, breast, vascular endothelium, heart, peritoneum, pancreas, colon, lung, kidney and liver). Each of these libraries contained > 20,000 tags. Since our KC library was relatively small compared to the public libraries, normalization may result in an artificial overrepresentation of the SAGE tags of low expressed genes, which may result in too high TPE values. This effect will not cause false positives (TPE value above the chosen threshold) for genes that are expressed in multiple tissues since there expression is too low. For genes that are expressed in few tissues a false positive could occur although one could argue that such genes would be tissue specific anyway.

Prior to determination of the TPE values for the SAGE library of interest, all tag counts that equal zero in any of the *N* SAGE libraries were replaced with 0.001 to allow the calculation of log-ratios. Expression ratios were determined for every tag with expression level *y_i_* in the SAGE library of interest with respect to expression levels in all other *N-1* libraries. Thus, *N-1* ratios were calculated, if *N* libraries were selected (including the library of interest). Every ratio *R_i_* was transformed to the log-ratio log(*R_i_*) and the mean log-ratio was calculated according to:$MLR=\frac{1}{N-1}\sum_{1}^{N-1} \log(Ri)$. The mean log-ratio measures the average overexpression or underexpression for a particular tag in the library of interest (gene). Subsequently, the number of SAGE libraries *B_i_* in which the tag is represented was determined. Since the mean log-ratio and *B_i_* generally have different ranges, the *B_i_* is scaled such that max(*B_i_*)=max(*MLR*). This ensures equal contributions of the MLR and *B_i_* to the TPE value. The tissue preferential expression is then calculated as $TPE=\sqrt{MLR^{2}+(N-Bi)^{2}}$, which is simply the length of the vector in the coordinate system defined by MLR and *B_i_* . After calculation, the TPE values were ranked according their value. Large positive TPE values represent tissue specific genes that are overexpressed in the library of interest. Tags with TPE values of <4 were excluded from further analysis since these tags occur very frequently in various cell types. Expression of tags with TPE values of ≥9 are indicative for tissue preferential expression in the epithelial cells used in this analysis. This threshold is chosen very high to prevent false positives and also diminishes the number of false positives that result from the normalization procedure.
